# Publisher Correction: Tropical explosive volcanic eruptions can trigger El Niño by cooling tropical Africa

**DOI:** 10.1038/s41467-017-01769-w

**Published:** 2017-11-15

**Authors:** Myriam Khodri, Takeshi Izumo, Jérôme Vialard, Serge Janicot, Christophe Cassou, Matthieu Lengaigne, Juliette Mignot, Guillaume Gastineau, Eric Guilyardi, Nicolas Lebas, Alan Robock, Michael J. McPhaden

**Affiliations:** 1 Laboratoire d’Océanographie et du Climat: Expérimentations et approches numériques, Sorbonne Universités, UPMC Université Paris 06, IPSL, UMR CNRS/IRD/MNHN, F-75005 Paris, France; 2 Indo-French Cell for Water Sciences, IISc-NIO-IITM-IRD Joint International Laboratory, NIO, Goa, 403002 India; 3 CECI, CNRS, Cerfacs, Université de Toulouse, Toulouse, 31057 France; 4 NCAS-Climate, University of Reading, Reading, RG6 6BB UK; 5 Department of Environmental Sciences, Rutgers University, New Brunswick, NJ 08901 USA; 6 Pacific Marine Environmental Laboratory (PMEL), NOAA, Seattle, WA 98115 USA

*Nature Communications* **8**:778 doi:10.1038/s41467-017-00755-6; Article published online: 3 October 2017

An incorrect version of the Supplementary Information was inadvertently published with this Article, which included proofing notes. The HTML has now been updated to include the correct version of the Supplementary Information.

