# Author Correction: Tropical explosive volcanic eruptions can trigger El Niño by cooling tropical Africa

**DOI:** 10.1038/s41467-018-03365-y

**Published:** 2018-02-22

**Authors:** Myriam Khodri, Takeshi Izumo, Jérôme Vialard, Serge Janicot, Christophe Cassou, Matthieu Lengaigne, Juliette Mignot, Guillaume Gastineau, Eric Guilyardi, Nicolas Lebas, Alan Robock, Michael J. McPhaden

**Affiliations:** 1 Laboratoire d’Océanographie et du Climat: Expérimentations et approches numériques, Sorbonne Universités, UPMC Université Paris 06, IPSL, UMR CNRS/IRD/MNHN, F-75005 Paris, France; 2 Indo-French Cell for Water Sciences, IISc-NIO-IITM-IRD Joint International Laboratory, NIO, 403002 Goa, India; 3 CECI, CNRS, Cerfacs, Université de Toulouse, 31057 Toulouse, France; 4 NCAS-Climate, University of Reading, RG6 6BB Reading, UK; 5 Department of Environmental Sciences, Rutgers University, New Brunswick, NJ 08901 USA; 6 Pacific Marine Environmental Laboratory (PMEL), NOAA, Seattle, WA 98115 USA

Correction to: *Nature Communications* 10.1038/s41467-017-00755-6, published online 03 October 2017

The original version of this Article omitted a reference to previous work in ‘Mann, M.E., Cane, M.A., Zebiak, S.E., Clement, A., Volcanic and Solar Forcing of the Tropical Pacific Over the Past 1000 Years, *J. Climate* **18**, 447–456 (2005)’. This has been added as reference 62 at the end of the fourth sentence of the fourth paragraph of the Introduction: ‘Early studies using simple coupled ocean–atmosphere models^26^ proposed that following volcano-induced surface cooling, upwelling in the eastern equatorial Pacific acting on a reduced vertical temperature contrast between the ocean surface and interior leads to anomalous warming in this region, thereby favouring El Niño development the following year^12,27,62^.’ This has been corrected in the PDF and HTML versions of the Article.

