# Supplementary material for: Tropical explosive volcanic eruptions can trigger El Niño by cooling tropical Africa
Source: Nat Commun. 2017 Oct 3;8:778. doi: 10.1038/s41467-017-00755-6 (PMC5626740; doi:10.1038/s41467-017-00755-6)
Supplement: Supplementary file 1 — Supplementary Information Supplementary Figures and Supplementary Tables [file 41467_2017_755_MOESM1_ESM.pdf]

Modelling centre	CMIP5 model(s)	Ensemble size	External forcing
GFDL	GFDL-CM3	5	GHG,SA,Oz,LU,Sl,Vl,SS,BC,MD,OC (GHG includes CO_2, CH_4, N_2O, CFC11, CFC12, HCFC22, CFC113)
	GFDL-CM2p1	10	GHG,SD,Oz,Sl,Vl,SS,BC,MD,OC (GHG includes CO_2, CH_4, N_2O)
	GFDL-ESM2M	1	GHG,SD,Oz,LU,Sl,Vl,SS,BC,MD,OC (GHG includes CO_2, CH_4, N_2O, CFC11, CFC12, HCFC22, CFC113)
	GFDL-ESM2G	1	GHG,SD,Oz,LU,Sl,Vl,SS,BC,MD,OC (GHG includes CO_2, CH_4, N_2O, CFC11, CFC12, HCFC22, CFC113)
CCCma	CMCC-CESM	1	Nat,Ant,GHG,SA,Oz,Sl
	CMCC-CMS	1	Nat,Ant,GHG,SA,Oz,Sl
CNRM	CNRM-CM5	10	GHG, SA, Sl, Vl, BC, OC
	CNRM-CM5-2	1	GHG, SA, Sl, Vl, BC, OC
MIROC	MIROC4h	0	GHG, SA, Oz, LU, Sl, Vl, SS, Ds, BC, MD, OC
	MIROC-ESM	3	GHG, SA, Oz, LU, Sl, Vl, MD, BC, OC
	MIROC5	5	GHG, SA, Oz, LU, Sl, Vl, SS, Ds, BC, MD, OC
CSIRO	CSIRO-Mk3-6-0	10	Ant,Nat (all forcings)
MOHC	HadCM3	10	GHG, Oz, SA, Sl, Vl, (GHG = CO_2, N_2O, CH_4, CFCs)
GISS	GISS-E2-H	6	GHG, LU, Sl, Vl, BC, OC, SA, Oz (also includes orbital change - BC on snow - Nitrate aerosols)
	GISS-E2-R	6	GHG, LU, Sl, Vl, BC, OC, SA, Oz (also includes orbital change - BC on snow - Nitrate aerosols)
NCAR	CCSM4	6	Sl GHG Vl SS Ds SD BC MD OC Oz AA LU
IAP	FGOALS-g2	4	GHG,Oz,SA,BC,Ds,OC,SS,Sl,Vl (GHG includes CO_2,CH_4,N_2O,CFC11,effective CFC12. Aerosol also includes sulfate.)
	FGOALS-s2	3	GHG, SD, Oz, Sl, Vl, SS, Ds, BC, OC
NCC	NorESM1-ME	1	GHG, SA, Oz, Sl, Vl, BC, OC
	NorESM1-M	3	GHG, SA, Oz, Sl, Vl, BC, OC
MPI	MPI-ESM-LR	3	GHG Oz SD Sl Vl LU
	MPI-ESM-MR	3	GHG,Oz,SD,Sl,Vl,LU
	MPI-ESM-P	2	GHG Oz SD Sl Vl LU
CCCma	CanCM4	0	GHG,Oz,SA,BC,OC,LU,Sl,Vl (GHG includes CO_2,CH_4,N_2O,CFC11,effective CFC12)
	CanESM2	5	GHG,Oz,SA,BC,OC,LU,Sl,Vl (GHG includes CO_2,CH_4,N_2O,CFC11,effective CFC12)
BCC	BCC-CSM1-1	3	Nat Ant GHG SD Oz Sl Vl SS Ds BC OC
	BCC-CSM1-1-m	3	Nat Ant GHG SD Oz Sl Vl SS Ds BC OC

Supplementary Table 1. CMIP5 1850-2005 historical simulations[31] used in this study, together with a description of the prescribed external forcing (using CMIP5 official abbreviated descriptors). Nat: natural forcing (for example, solar and volcanic). **LU**: land-use change. **Ant**: anthropogenic forcing (a mixture, for example, well-mixed greenhouse gases, aerosols, ozone, and land-use changes). **Sl**: solar irradiance. **GHG:** well-mixed greenhouse gases (a mixture, not explicitly defined here). **Vl**: volcanic aerosol. **SD:** anthropogenic sulphate aerosol, accounting only for direct effects. **SS**: sea salt. **SI**: anthropogenic sulphate aerosol, accounting only for indirect effects. **Ds**: Dust SA (= SD + SI) anthropogenic sulphate aerosol direct and indirect effects. **BC**: black carbon. **TO**: tropospheric ozone. **MD**: mineral dust. **SO**: stratospheric ozone. **OC**: organic carbon. **Oz** (= TO + SO): ozone (= tropospheric and stratospheric ozone). **AA**: anthropogenic aerosols (a mixture of aerosols, not explicitly defined here).

Experiment Name	Origin of ocean boundary condition	Origin of land boundary condition	External forcing in the atmosphere
Control	Control	Control	No volcano
ALL	Pinatubo	Pinatubo	Volcano
OCEAN	Pinatubo	Control	No volcano
ATM	Control	Control	Volcano
LAND	Control	Pinatubo	No volcano
LAND-T	Control	Pinatubo 25°N-25°N and control elsewhere	No volcano
LAND-ET	Control	Pinatubo 25°N-90°N; 90°S-25°S and control elsewhere	No volcano
LAND-SEA	Control	Pinatubo 90°E-160°W; 10°N-30°N and control elsewhere	No volcano
LAND-MC	Control	Pinatubo 90°E-150°W; 10°S-10°N and control elsewhere	No volcano
LAND-Africa	Control	Pinatubo 15°W-50°E; 10°S-30°N and control elsewhere	No volcano

Supplementary Table 2. Overview of sensitivity simulations performed with the atmospheric component of the IPSL-CM5B model used in this study and their boundary conditions. *Control* means boundary conditions that resulted from a free-running control ensemble of the coupled model experiment imposed as fixed boundary conditions for atmosphere-only simulations. *Pinatubo* means boundary conditions that resulted from a free-running 1991 Pinatubo experiment imposed as fixed boundary conditions for atmosphere-only simulations.

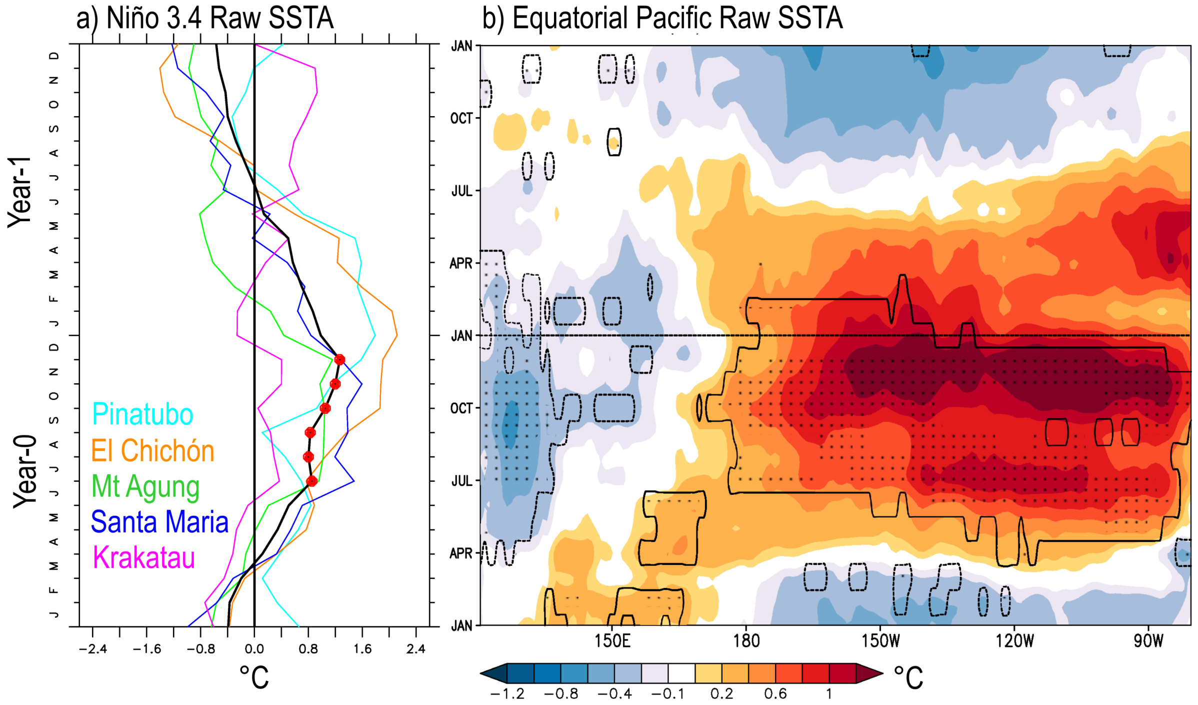

Supplementary Figure 1. Observed Sea Surface Temperature response to the five main eruptions with stratospheric aerosol injection during 1870-2010. a Evolution of the composited Niño 3.4 (5°S-5°N, 170°W-120°W) raw SST anomalies (SSTA, in °C) over a two year period following January of the five largest tropical explosive volcanic years of the 1870-1999 period in HadISST observations[46] (black line). The small red dots denote when the 5 individual events have a consistent sign anomaly. **b** Same as **a** but shown as a longitude-time section. Stippling highlights times and locations for which the 5 eruptions have consistent sign anomalies and the contour corresponds to the 90% confidence level of this anomaly according to a two-tailed Student *t*-test. In both panels, the composited anomalies are relative to the preceding 5-year climatology. This figure is similar to Fig 1a and b but using raw SST anomalies instead of relative SST anomalies.

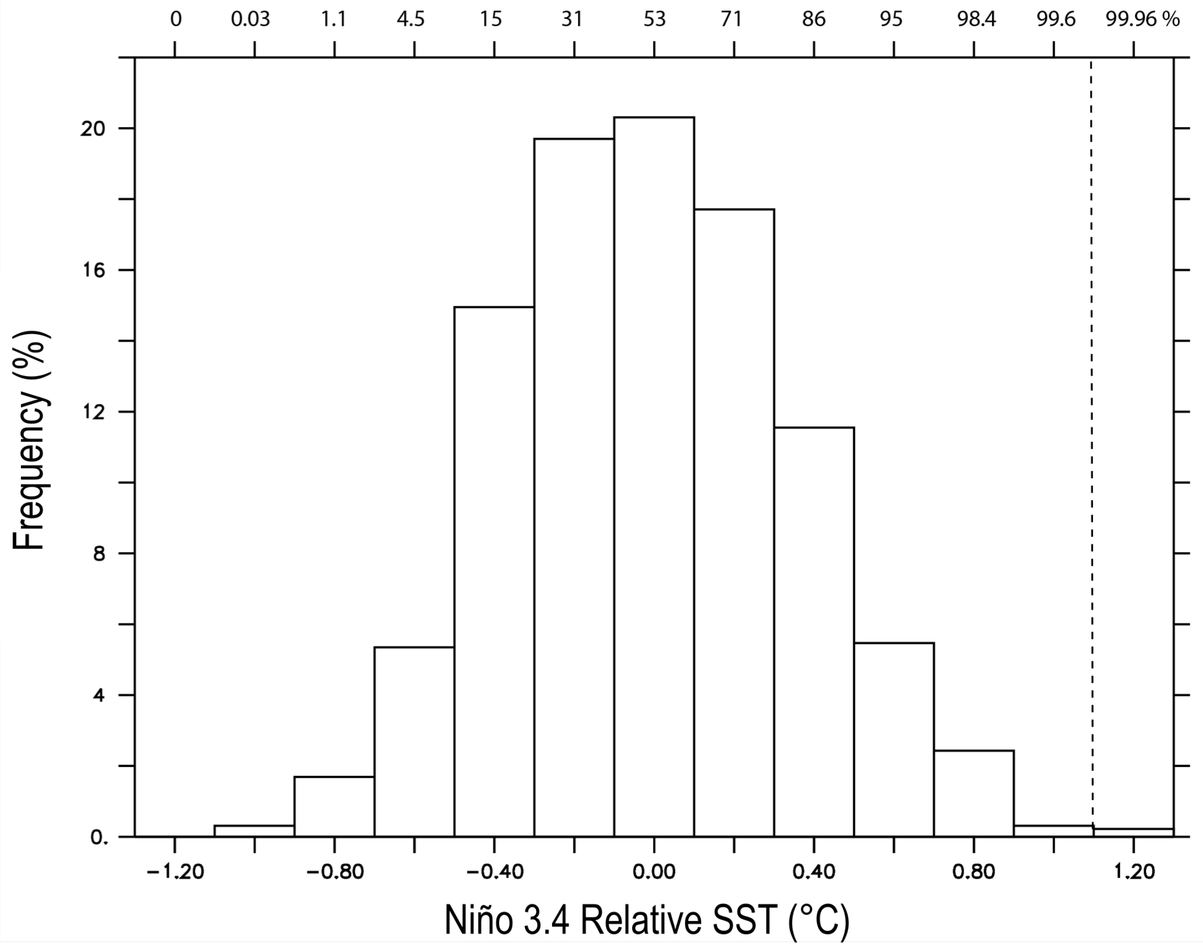

Supplementary Figure 2. Probability density function of the composite November-December-January Niño 3.4 average relative Sea Surface Temperature anomaly for five randomly-selected years in HadISST observations[46]. The upper abscissa label corresponds to the probability that the composite value exceeds that of the lower abscissa. The Probability density function (PDF) is built using a Monte-Carlo method based on randomly picking 5 unique years in the 1870-1999 period 10 000 times. The vertical dotted line indicates the composite value of the November-December-January (NDJ) Niño 3.4 relative Sea Surface Temperature (SST in °C) following the five largest tropical explosive volcanic events during the twentieth century (1870–1999). There is only a 0.3% chance that 5-randomly picked years have a composite NDJ Nino3.4 relative SST anomaly that exceeds the observed value after the 5 largest tropical eruptions over the historical period.

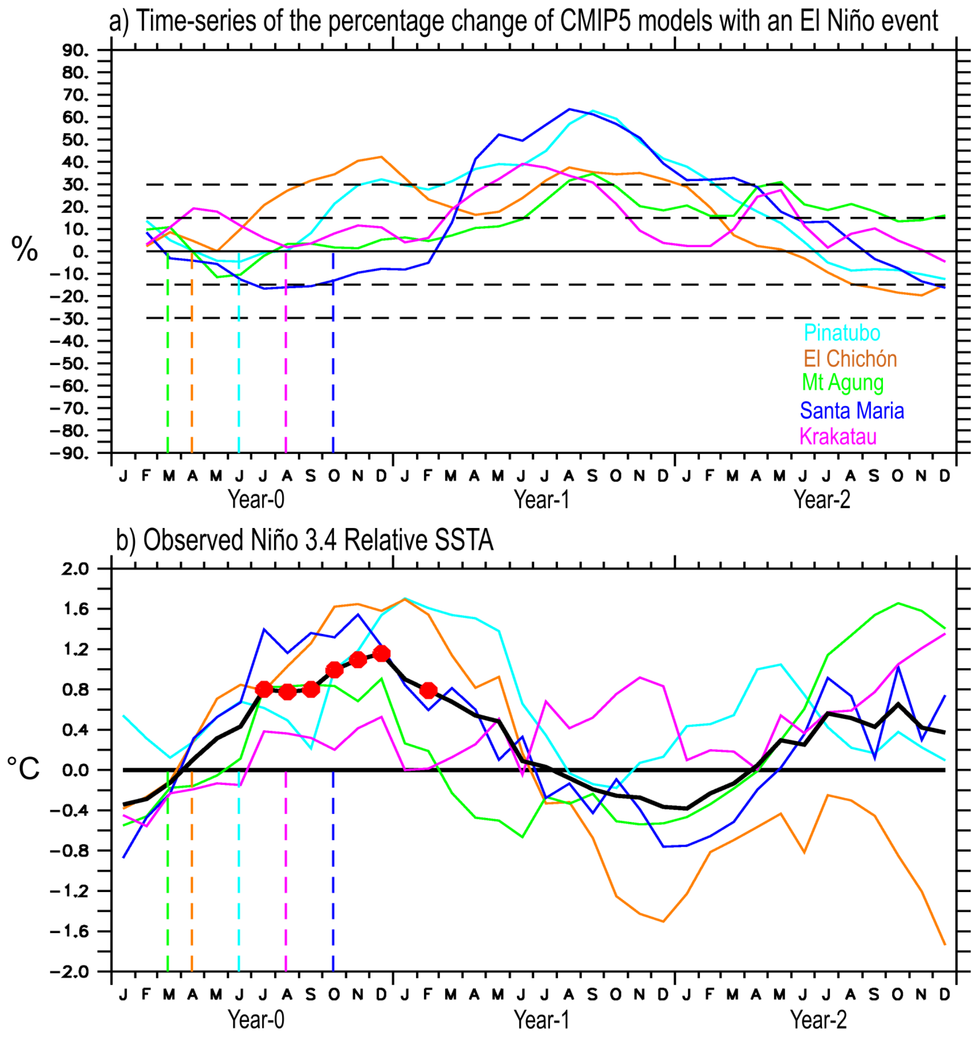

Supplementary Figure 3. CMIP5 model simulations and observed relative Sea Surface Temperature response to explosive tropical volcanism over a two years period following the year of the five largest tropical explosive volcanic years over the 1870-1999 period. a Multi-model averaged composite of the percentage of El Niño occurrence probability relative to the climatological El Niño probability in CMIP5 historical simulations. El Niño events are defined as relative SST anomaly (SSTA) in Niño3.4 exceeding 1/2 standard deviation. **b** Evolution of the composited Niño 3.4 (5°S-5°N, 170°W-120°W) relative SSTA (in °C) in HadISST observations[46]. Niño3.4 relative SSTA are computed relative to the preceding 5 years baseline climatology. The black line is the average of the 5 eruptions. The small red dots denote that the 5 individual events (colour lines) have a consistent sign anomaly. The vertical dotted lines localize the eruption date for Pinatubo (June 1991, light blue -lines), El Chichón (April 1982, orange-lines), Mt Agung (March 1963, green lines), Santa María (October 1902, dark blues), and Krakatau (August 1883, pink line). Panel b is the same as Figure 1a, but over a three-year period to allow comparison with the CMIP5 model results.

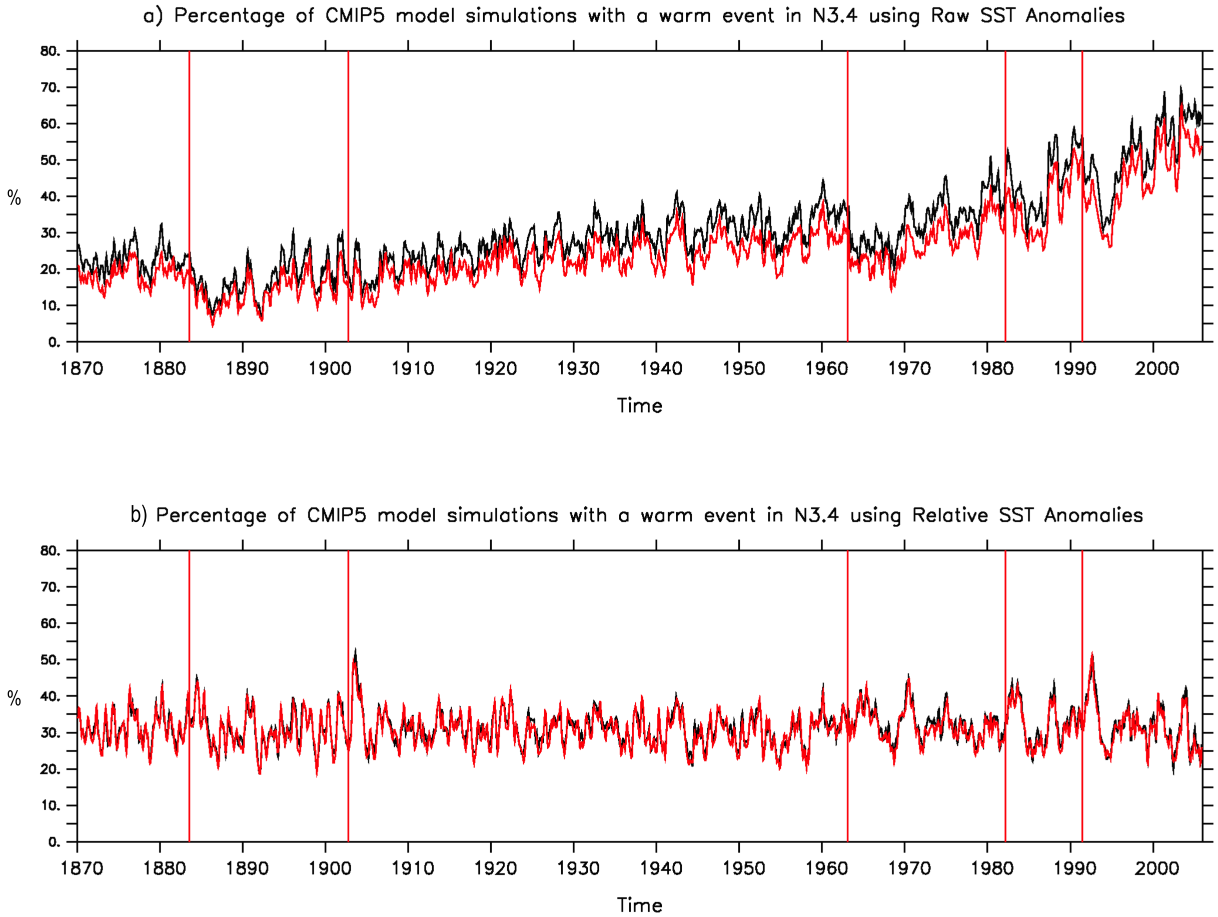

Supplementary Figure 4. Percentage of El Niño occurrence computed from CMIP5 historical simulations. El Niño events are defined as positive **a** raw and **b** relative Sea Surface Temperature (SST) anomaly in Niño3.4 exceeding 1/2 standard deviation. Niño3.4 SST anomalies are computed relative to both the 1961-1990 (red curve) and the 1870–2005 baseline climatology (black curve). Relative SST anomalies are defined as SST anomalies minus their average over the entire 20°N-20°S band. The vertical red lines localize each eruption date.

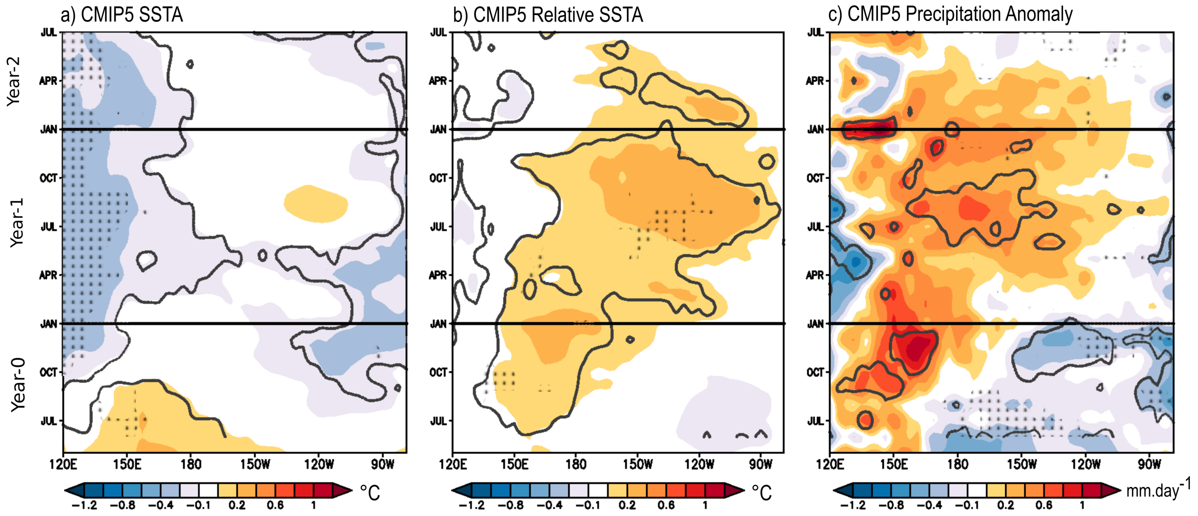

Supplementary Figure 5. Equatorial Pacific response following the Pinatubo eruption in CMIP5 historical simulations. Zonal-time section of the Pacific (120°E-80°W) 5°N-5°S CMIP5 simulations mean for **a** raw Sea Surface Temperature anomalies (SSTA, °C), **b** relative SST anomalies (°C) and **c** precipitation anomalies (mm day^{-1}) in response to the Pinatubo eruption. Time runs from June during the year of the Pinatubo eruption (Year 0) to July 2 years later, for the ensemble mean of 106 CMIP5 historical simulations. Stippling indicates time and locations for which more than two-thirds of the members have anomalies of consistent sign. Contours indicate statistically significant anomalies at the 90% confidence level, based on a two-tailed Student *t*-test. Relative SST anomalies are defined as SST anomalies minus their average over the entire 20°N-20°S band.

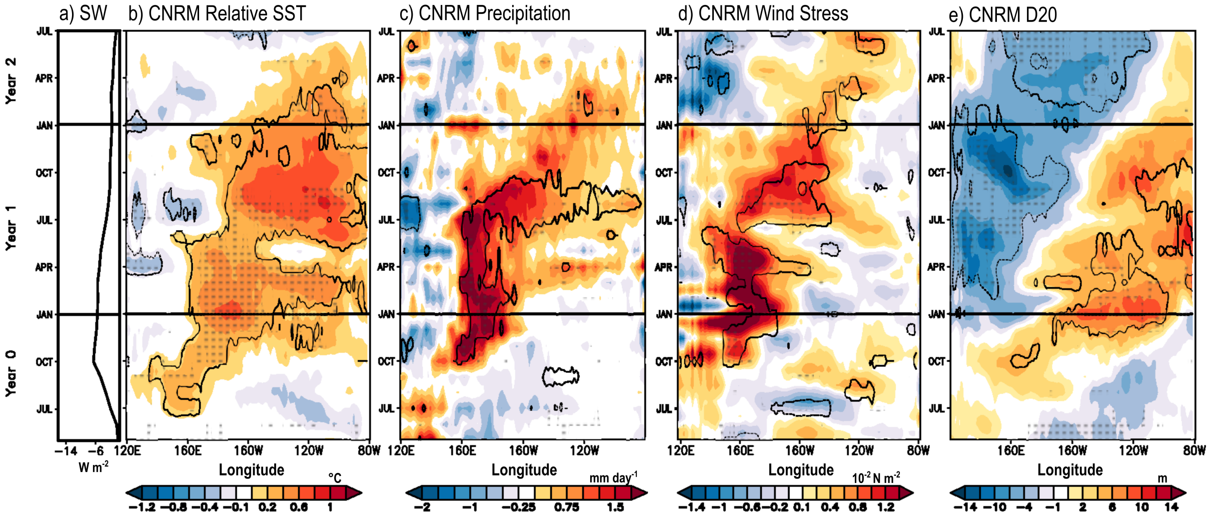

Supplementary Figure 6. Equatorial Pacific response following the Pinatubo eruption in CNRM-CM5 Pinatubo experiments. Clear-sky surface shortwave radiations, relative Sea Surface Temperature (SST), precipitation, zonal wind stress, and 20°C isotherm depth changes over the Pacific area (120°E-80°W) following the Pinatubo eruption in CNRM-CM5[50] sensitivity experiments. The ensemble consists of 23 members sampling random initial conditions in the unforced historical and control run (see text and Supplemental Information). **a** Time-series of mean tropical (20°S-20°N) surface clear sky shortwave. **b** Longitude–time section of 5°S–5°N anomalous relative SST (°C); **c** precipitation (mm day^{-1}), **d** zonal wind stress (10^{-2} N m^{-2}), and **e** 20°C isotherm depth (m) from June of the year of the Pinatubo eruption (Year 0) until October 2 years after (Year 2). Stippling indicates time and locations for which at least two thirds of the individual members (23 in total) display consistent sign anomalies. Contours indicate anomalies significant at the 90% confidence level, based on two-tailed Welch's *t*-test.

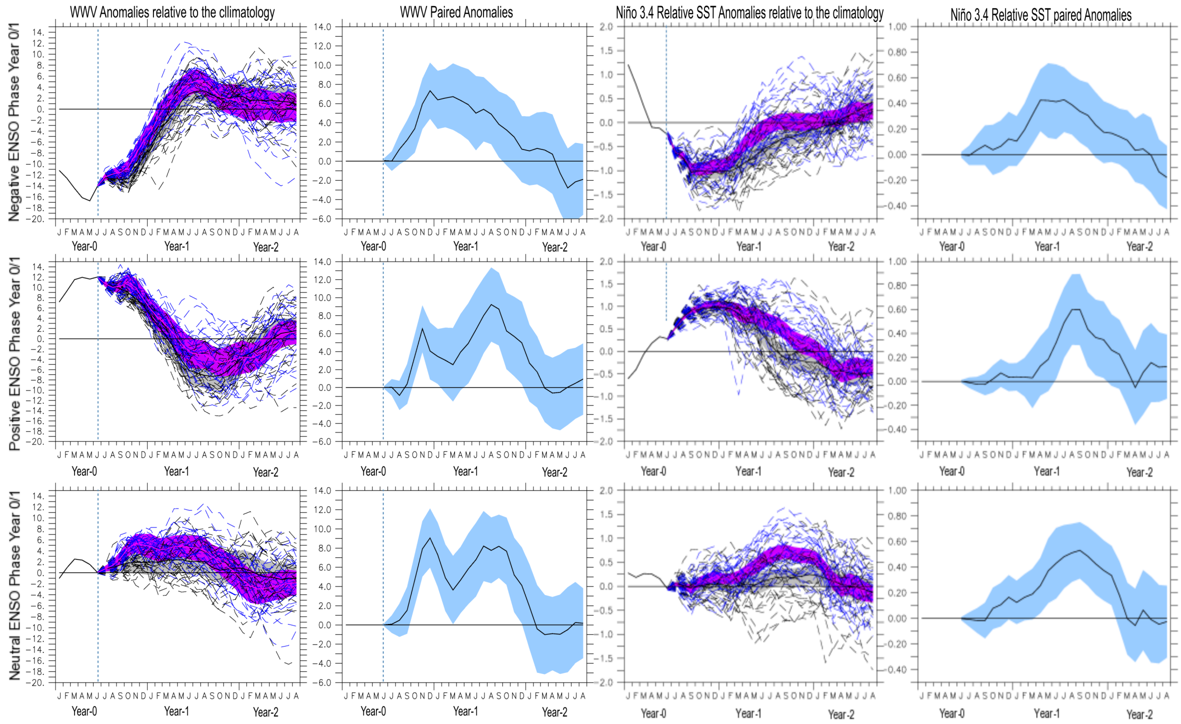

Supplementary Figure 7. Equatorial Pacific Warm Water Volume, Niño 3.4 Relative Sea Surface Temperature anomalies time series in the IPSL-CM5B three model ensembles (30 members). Warm Water Volume (WWV, in 10^{14} m^3) and Niño 3.4 (5°N-5°S, 170°W-120°W) Sea Surface Temperature (SST in °C) anomalies respectively to the climatology for the unforced (black dotted lines), forced members (blues dotted lines) and paired anomalies are shown for the ensemble starting from discharged WWV initial conditions (**top row**); the ensemble starting from recharged WWV initial conditions (**middle row**) and the ensemble starting from near neutral WWV initial conditions (**bottom row**). The colour shading on each panel illustrates how each ensemble is distributed around the mean (90% confidence interval on the two-tailed Welch's *t*-test). The vertical dashed line in each panel indicates the date of the eruption and the start time of each ensemble members. WWV is defined as the average 20°C isotherm depth (a good indicator of the thermocline depth in the tropics), within 5°S–5°N and 120°E–80°W, as in, for example, Meinen and McPhaden (2000)[4].

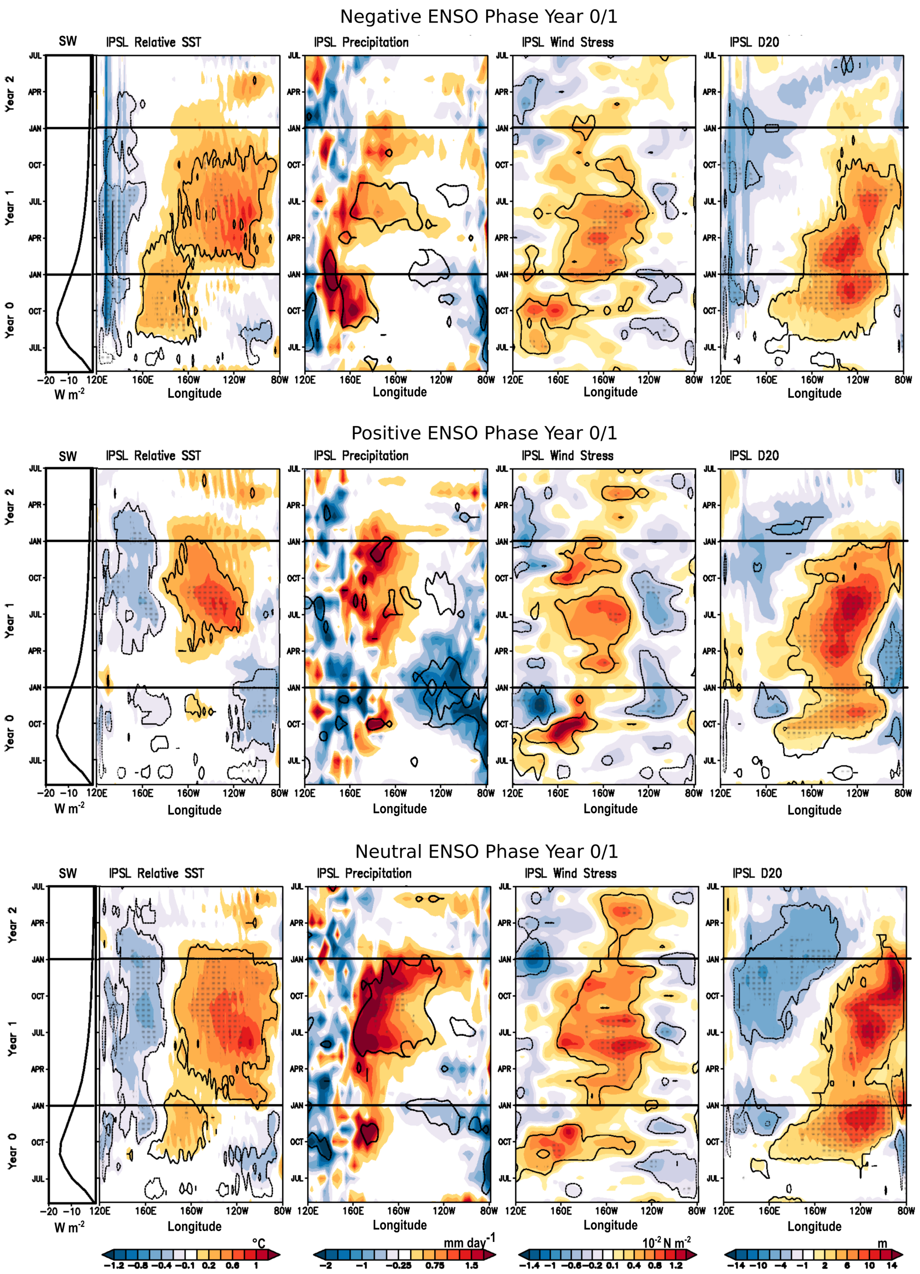

Supplementary Figure 8. Equatorial Pacific response following the Pinatubo eruption. Time-series of mean tropical (20°S-20°N) clear-sky surface shortwave radiation and longitude–time section of 5°S–5°N paired anomalous relative Sea Surface Temperature (°C), precipitation (mm day^{-1}), zonal wind stress (10^{-2} N m^{-2}), and 20°C isotherm depth (m) over the Pacific area (120°E-80°W) following the Pinatubo eruption in IPSL-CM5B experiments from June of the year of the Pinatubo eruption (Year 0) until July 2 years after (Year 2). Stippling indicates time and locations for which at least two thirds of the individual members (30 in total) display consistent sign anomalies for ocean-atmosphere conditions leading to **top row** La Niña, **central row** El Niño and **bottom row** neutral phase at the end year of the eruption year in the unforced control run based on the Warm Water Volume (see text and Methods). Contours indicate anomalies significant at the 90% confidence level, based on the two-tailed Welch's *t*-test.

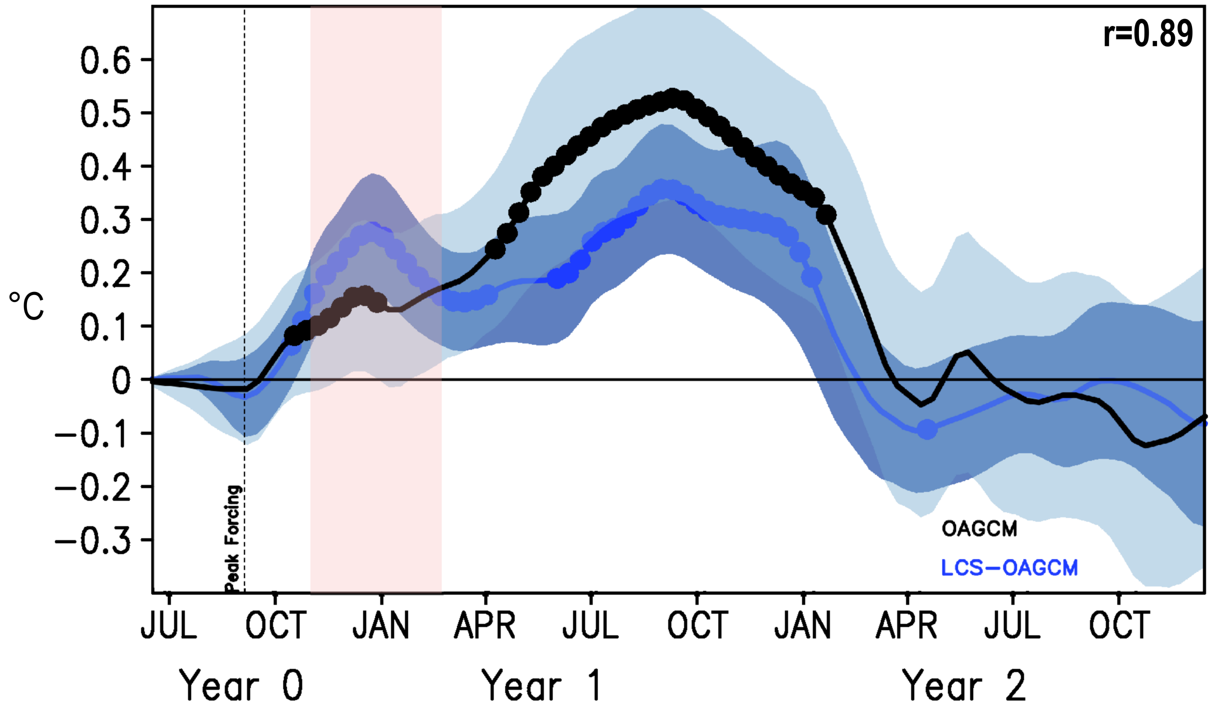

Supplementary Figure 9. Relative Sea Surface Temperature anomalies following the Pinatubo eruption. Niño 3.4 (5°N-5°S, 170°W-120°W) relative Sea Surface Temperature (°C) anomalies from the IPSL-CM5B coupled model ensemble (black) with initial conditions leading to neutral ENSO state at the end of the eruption year in the unforced control run. The blue curve indicates relative SST anomalies for the linear ocean model when it is forced by Pacific wind anomalies from the IPSL-CM5B coupled model simulations. See text for details. The dots indicate months for which at least two-thirds of the individual members display anomalies of consistent sign. The correlation (*r*) between both curves is given at the upper right of the panel.

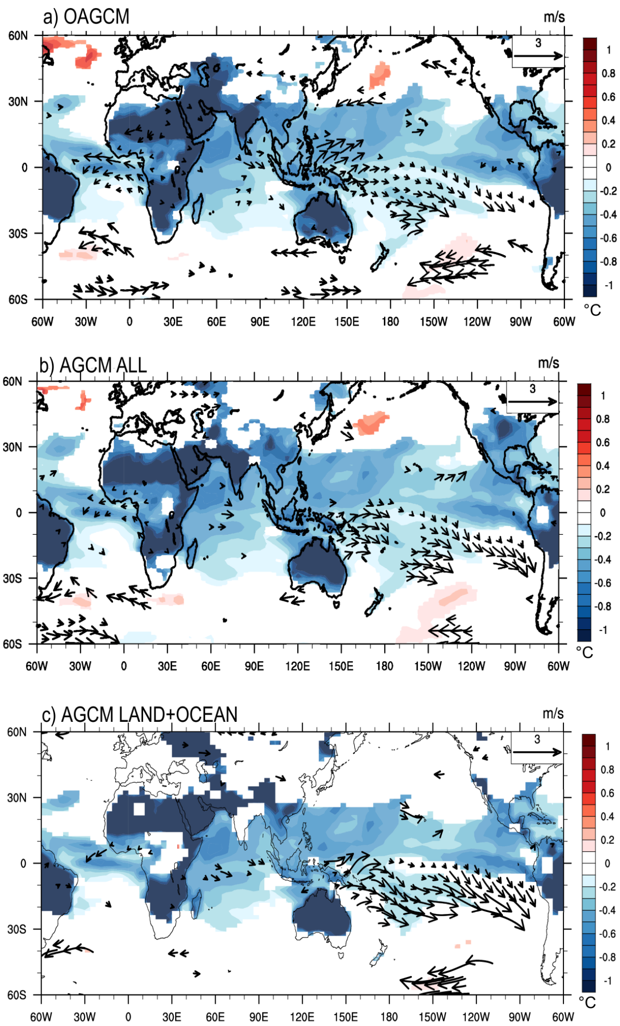

Supplementary Figure 10. Global surface temperature and wind patterns in response to the volcanic forcing. August-September-October of Year 0 mean surface temperature (°C) and surface wind (m s^{-1}, vectors) paired anomalies after the Pinatubo eruption in **a** the IPSL-CM5B coupled model ensemble starting from neutral conditions, and with its atmosphere component initialized with **b** ALL, **c** the sum of atmosphere component initialized with OCEAN and LAND boundary conditions. Anomalies are shown only when significant at 90% according to the two-tailed Welch's *t*-test.

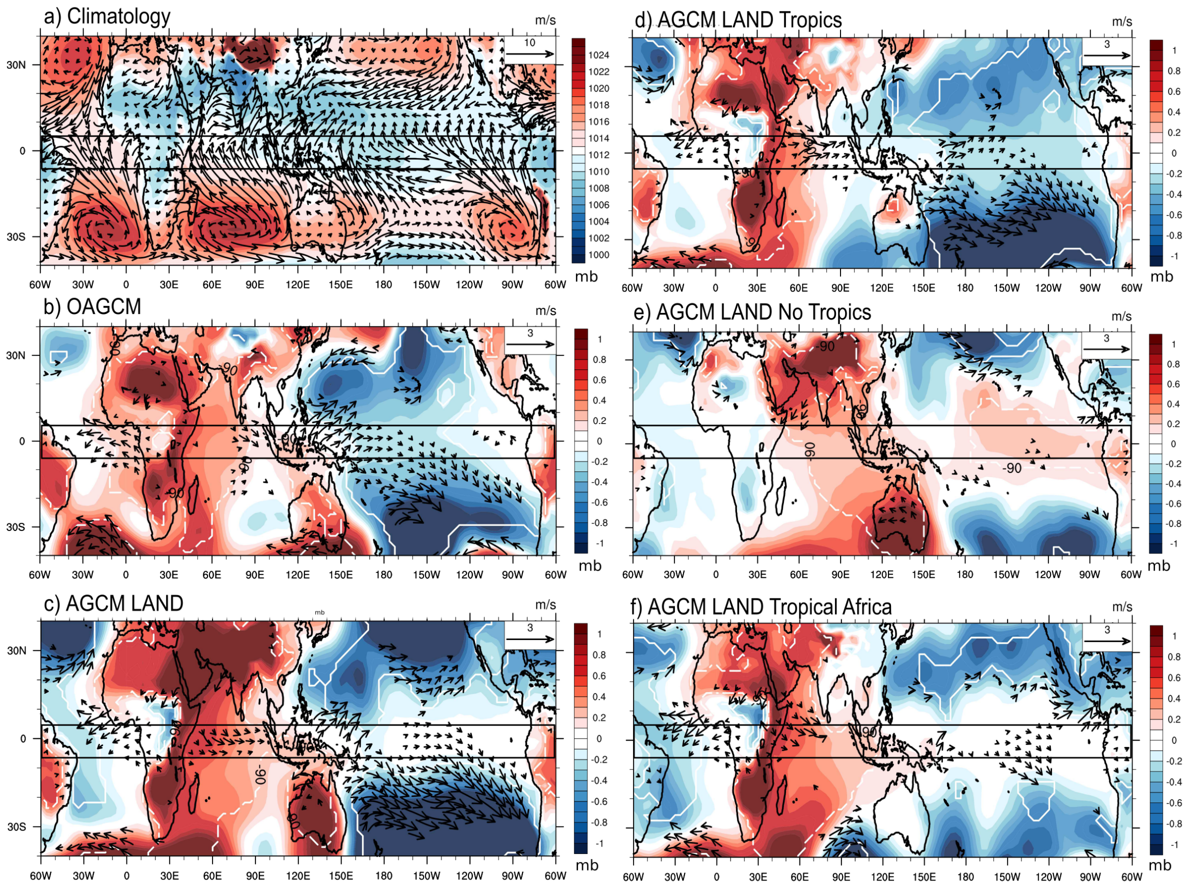

Supplementary Figure 11. Tropical sea level pressure and wind patterns in response to the volcanic forcing in August-September-October of Year-0. Mean sea level pressure (mb, shading) and surface wind (m s^{-1}, vectors) climatology in **a**, and paired anomalies after the Pinatubo eruption in **b** for the IPSL-CM5B coupled model ensemble starting from neutral conditions, and with its atmosphere component initialized with **c** LAND, **d** LAND tropical cooling only, **e** LAND with no tropical cooling only and **f** LAND tropical Africa cooling only boundary conditions. The white contours on sea level pressure anomalies indicate the significance level at 90% according to the two-tailed Welch's *t*-test. The surface wind anomalies are shown only when significant at 90% according to the two-tailed Welch's *t*-test.

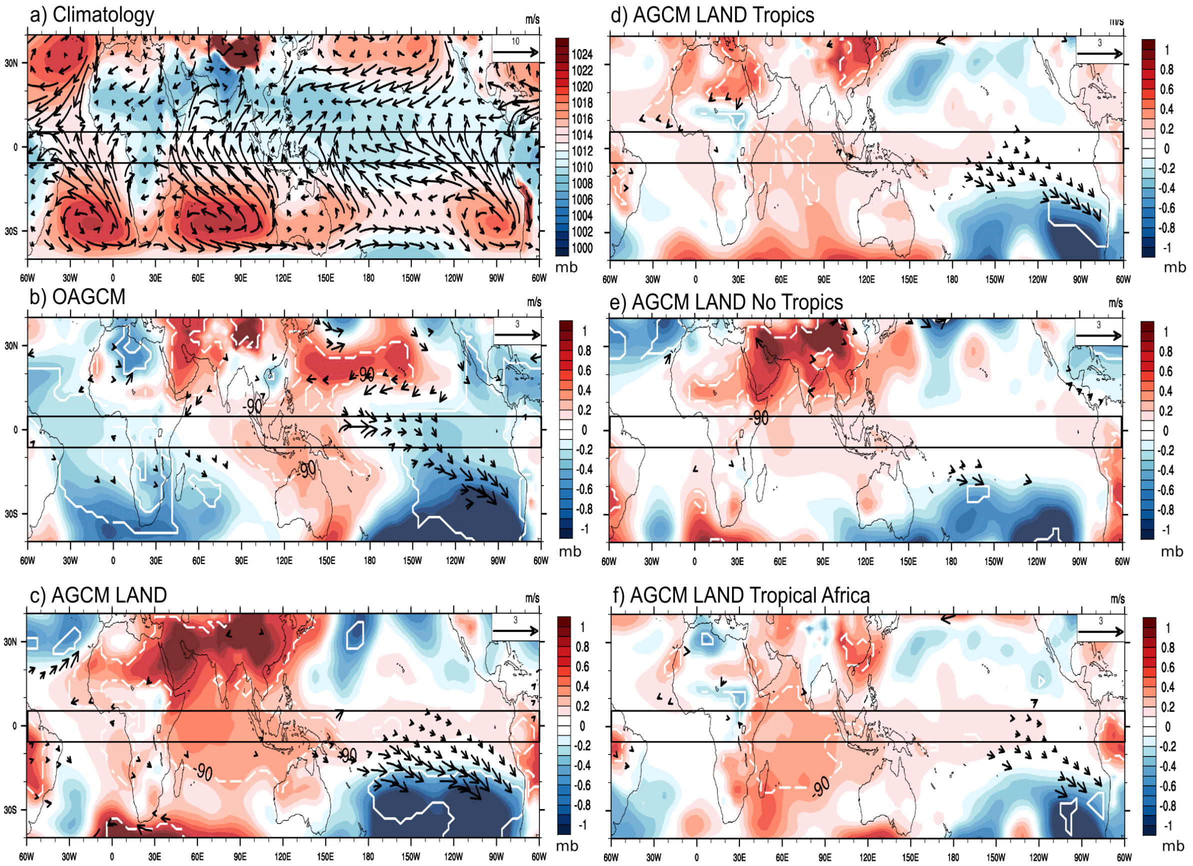

Supplementary Figure 12. Tropical sea level pressure and wind patterns in response to the volcanic forcing in August-September-October of Year-1. Mean sea level pressure (mb, shading) and surface wind (m s^{-1}, vectors) climatology in **a**, and paired anomalies after the Pinatubo eruption in **b** for the IPSL-CM5B coupled model ensemble starting from neutral conditions, and with its atmosphere component initialized with **c** LAND, **d** LAND tropical cooling only, **e** LAND with no tropical cooling only and **f** LAND tropical Africa cooling only boundary conditions. The white contours on sea level pressure anomalies indicate the significance level at 90% according to the two-tailed Welch's *t*-test. The surface wind anomalies are shown only when significant at 90% according to the two-tailed Welch's *t*-test.

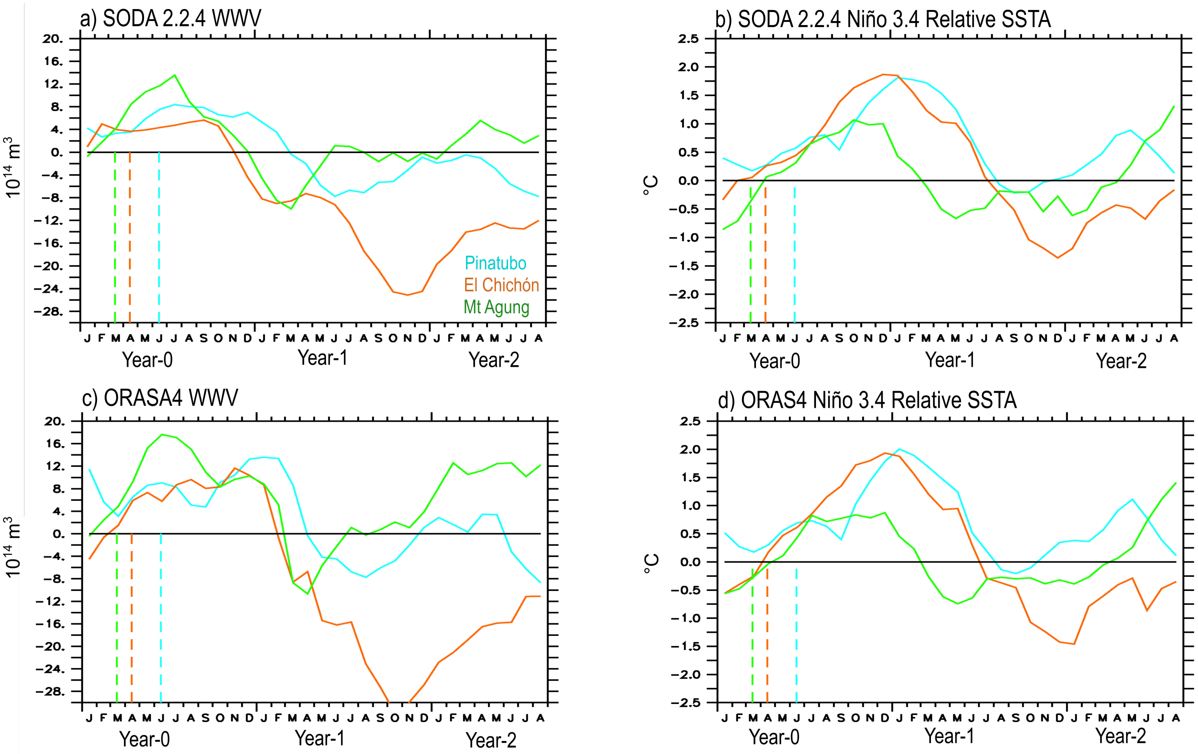

Supplementary Figure 13. Equatorial Pacific Warm Water Volume and Niño 3.4 (5°N-5°S, 170°W-120°W) relative Sea Surface Temperature anomalies in Ocean Reanalyses products. Time series of Equatorial Pacific Warm Water Volume (WWV, in 10^{14} m^3) and Sea Surface Temperature anomalies (SSTA in °C) over a two years period following January of the 3 largest tropical explosive volcanic years of the 1960-1999 period in SODA.v224[60] (top row) and ORAS4[61] (bottom row) Ocean Reanalyses products. The anomalies are computed relative to the preceding 5 years baseline climatology for each eruption. The vertical dotted lines localize the eruption date for Pinatubo (June 1991, light blue -lines), El Chichón (April 1982, orange-lines), Mt Agung (March 1963, green lines).
